# Evaluating the Development Status of Fluorescence-Guided Surgery (FGS) in Pediatric Surgery Using the Idea, Development, Exploration, Assessment, and Long-Term Study (IDEAL) Framework

**DOI:** 10.3390/children10040689

**Published:** 2023-04-05

**Authors:** Alessandra Preziosi, Irene Paraboschi, Stefano Giuliani

**Affiliations:** 1 Fondazione IRCCS Ca’ Granda Ospedale Maggiore Policlinico di Milano, 20122 Milano, Italy; 2 Cancer Section, Developmental Biology and Cancer Programme, UCL, Great Ormond Street Institute of Child Health, London WC1N 1EH, UK; 3 Department of Specialist Neonatal and Paediatric Surgery, Great Ormond Street Hospital NHS Foundation Trust, London WC1N 3JH, UK; 4 Wellcome/EPSRC Centre for Interventional and Surgical Sciences (WEISS), University College London, London W1W 7TY, UK

**Keywords:** fluorescence-guided surgery, pediatric surgery, IDEAL framework and recommendations, indocyanine green, fluorescence imaging

## Abstract

Fluorescence-guided surgery (FGS) is used in many pediatric subspecialties but there are currently no standard guidelines or outcome data. We aimed to assess the current status of FGS in pediatrics using the Idea, Development, Exploration, Assessment, and Long-term study (IDEAL) framework. Clinical papers on FGS in children published from January 2000 to December 2022 were systematically reviewed. The stage of research development was measured considering seven fields of application: biliary tree imaging, vascular perfusion for gastrointestinal procedures, lymphatic flow imaging, tumor resection, urogenital surgery, plastic surgery, and miscellaneous procedures. Fifty-nine articles were selected. For each field of application, the overall IDEAL stage was determined to be 2a for biliary tree imaging (10 publications, 102 cases), 1 for vascular perfusion for gastrointestinal procedures (8 publications, 28 cases), 1 for lymphatic flow imaging (12 publications, 33 cases), 2a for tumor resection (20 publications, 238 cases), 2a for urogenital surgery (9 publications, 197 cases), and 1-2a for plastic surgery (4 publications, 26 cases). One report did not belong to any categories. FGS in children is still in an early phase of adoption and development. We recommend using the IDEAL framework as a guide and suggest developing multicenter studies to define the standard guidelines, effectiveness, and outcomes.

## 1. Introduction

Fluorescence imaging is an emerging intraoperative technique used in a broad range of surgical applications (e.g., to visualize tissue perfusion, vascularization, lymphatic flow, biliary tree anatomy, and tumors).

Recently there has been an increasing use of fluorescence-guided surgery (FGS) in several fields of pediatrics. However, as a surgical innovation, there is still a lack of guidelines for the use of FGS in children.

**Figure 1:**
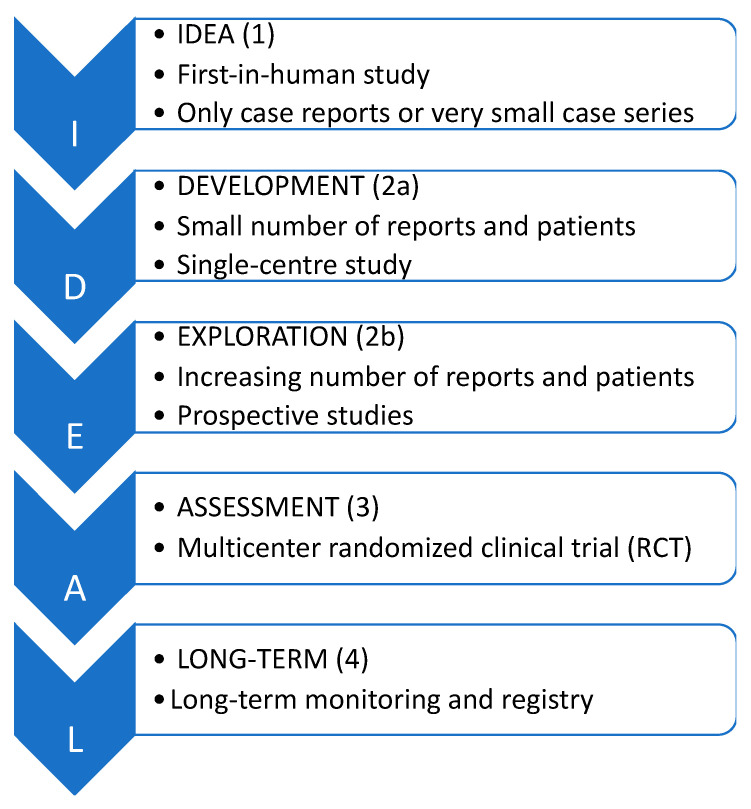
The IDEAL staging system.

The assessment of innovative procedures is always a challenging process as the evolution of the technique itself, the surgeons’ learning curves, and the risk of subjective procedural quality are factors that need to be considered. The identification of these difficulties has led to the creation of the Idea, Development, Exploration, Assessment, and Long-term follow-up (IDEAL) framework that describes five stages of assessment. Each of these stages is based on the types of articles already published in which the technique was used (Figure 1) [1,2,3].

In 2021, in response to the widening use of FGS, Ishizawa et al. used the IDEAL framework to identify the state of research for the use of fluorescence imaging across surgical fields and applications in adults [1].

We decided to perform a systematic review of the current applications of FGS in pediatrics using the IDEAL framework, to assess its current status and suggest further developments in this field.

## 2. Material and Methods

This systematic review was made according to the Preferred Reporting Items for Systematic Reviews and Metanalysis Statement (http://www.prisma-statement.org/ accessed on 20 September 2022).

A broad search was carried out in the electronic database MEDLINE from January 2000 to December 2022 using combinations of keywords such as “Fluorescent-guided” [All Fields] OR “Fluorescence-guided” [All Fields] AND “Surgery” [All Fields]. Further records were identified by hand-searching the references cited in each article. Two researchers conducted data extraction and quality assessment independently.

The inclusion criteria were original studies; written in English; reporting only cases of fluorescence imaging for the surgical treatment of pediatric patients (0–18 years old) affected by thoracic, abdominal, and urogenital diseases, malformations, or tumors. Preclinical studies, papers not reporting own clinical experience, and studies employing fluorescent dyes for diagnostic reasons only and/or describing neurosurgical, vascular, ophthalmological, otolaryngological, and maxillofacial surgical procedures were excluded.

The selected articles were classified according to their field of application in seven categories: biliary tree imaging, vascular perfusion for gastrointestinal (GI) procedures, lymphatic flow imaging, tumor resection, urogenital surgery, plastic surgery, and miscellaneous procedures in case the reports did not belong to any of the previous categories.

Each article selected was assessed by using the IDEAL framework, as previously described (https://www.ideal-collaboration.net/the-ideal-framework accessed on 20 September 2022).

The IDEAL stage for each field of application was determined by calculating the mode from the papers collected in the specific category.

## 3. Results

**Figure 2:**
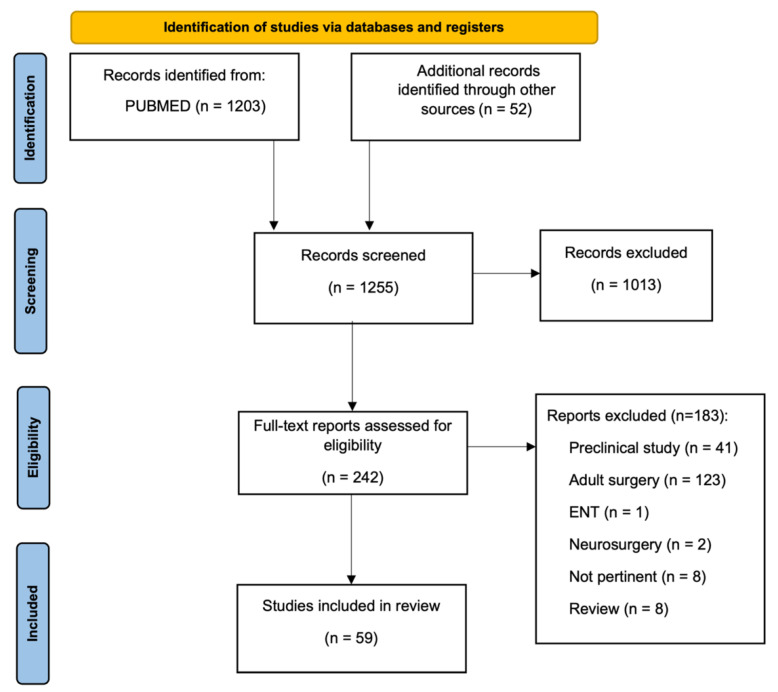
PRISMA flowchart. Abbrev. ENT: otolaryngology.

As shown in the PRISMA flow diagram (Figure 2), 1228 papers were screened. In total, 799 were excluded on a title basis and 214 were excluded on an abstract basis such that 242 studies were evaluated on a full-text basis. Out of these, 183 were excluded because they were either focused on the adult population (*n* = 123), they were preclinical studies (*n* = 41), review articles (*n* = 8), articles describing otolaryngology (*n* = 1), neurosurgery procedures (*n* = 2), or the topic was not pertinent (*n* = 8). Overall, 59 studies were included in the systematic review, reporting on 558 patients in total.

Out of the 59 studies included in the final analysis, 7 (12%) articles focused on the real-time imaging of the biliary tree only, 8 (14%) on the vascular perfusion for GI procedures only, 12 (20%) on lymphatic flow only, 18 (30%) on tumor resections only, 6 (10%) on urogenital surgery only, and 4 (7%) on plastic surgery only. Three (5%) papers described several indications in the same case series, including urogenital surgery, biliary tree imaging, and tumor resection. One case report (2%) did not belong to any of these previous groups as the dye was administered via the mouth to assess the patency of the duodenum in a neonate with a duodenal web.

Three dyes were used in these studies: indocyanine green (ICG) was the most used dye (58 out of 59 studies), whereas fluorescein sodium and methylene blue were used in only one study each.

Focusing on imaging devices, the fluorescence signal was detected by different optical systems marketed by: Karl Storz in 14/59 (23%), Stryker in 11/59 (18%), Hamamatsu Photonics in 11/59 (18%), Firefly/Intuitive in 4/4 applications with robotic surgery, Medtronic in 5/59 (8%), Zeiss in 1/59 (1.7%), Sony in 1/59 (1.7%), Mizuho Medical Co. in 1/59 (1.7%), Panasonic AVC Networks Company in 1/59 (1.7%), and Zhuhai Dipu Medical Technology Co. in 1/59 (1.7%). Twelve articles (20%) did not disclose the imaging system employed.

### 3.1. Current Applications and the IDEAL Stage for Each Category

#### 3.1.1. Biliary Tree Imaging

Our search yielded 10 eligible studies focusing on biliary tree imaging. Out of these studies, 2 were case reports, 7 were retrospective studies, and 1 was a prospective study. In total, 102 cases were published in this field with the highest number of patients in any study being 31. (Table 1)

Seven papers described the use of ICG for guiding pediatric cholecystectomy (*n* = 86) [4,5,6,7,8,9,10]. Two studies explained the application of FGS during Kasai procedures (*n* = 15) [11,12]. One case report employed the use of ICG for the visualization of biliary leakage after hepatectomy [13]. Intravenously injected ICG was the dye used in each study. 

A dose of 0.4 mg/kg was the most commonly used to identify the biliary structures for laparoscopic cholecystectomies (range from 0.1 to 0.5 mg/kg) [4,8,10]. The median timing of administration was 15 h preoperatively. Only in two papers was ICG directly injected intravenously in the operating room before trocar placement for laparoscopic cholecystectomies [7,9]. Although Esposito et al. supported the preoperative injection to avoid fluorescence from the liver parenchyma [8], the different times of injection did not affect the usefulness of ICG [7,9]. Considering the effectiveness indeed, Esposito et al. stated that the intraoperative ICG visualization rate was 95.2% during cholecystectomies [5]. Only one study reported that ICG did not provide adequate information for dissection in a case of gallbladder duplication [6]. No adverse or allergic reactions were reported.

Considering the application of fluorescence imaging during Kasai procedures, there is no consensus about the dose administered: 0.5 mg/kg of ICG was injected by Yanagi et al. and 0.1 mg/kg of ICG was administered by Hirayama et al. In both cases, ICG was intravenously injected 24 h before surgery [11,12], proving to be particularly beneficial for the delicate dissection of the proximal biliary tree in biliary atresia. Yanagi et al. observed that the fluorescence arising from the dissected plane at the porta hepatis helped the surgeon to visualize biliary juice leaking from the raw liver in 80% of cases [11]. No adverse or allergic reactions were reported in any studies.

Even though FGS applied to biliary tree surgery is safe and effective, the lack of multicenter studies, prospective cohort studies, and randomized controlled trials (RCTs) suggest that the level of IDEAL is only Stage 2a.

Certainly, prospective cohort studies are now needed to assess the advantages of fluorescence in biliary tree imaging compared to the standard technique.

**Table 1:** Summary for biliary tree imaging.

Authors	Year	No. of Procedures Performed	Mean pt Age	Indication	Surgical Procedure	Dose of Dye	Imaging System	Study Design	IDEAL Framework Stage
Esposito et al. [5]	2022	21	median 12.2 y	Cholelithiasis	Laparoscopic cholecystectomy	ICG 0.35 mg/kg iv	Karl Storz	Retrospective study	2a
Esposito et al. [10]	2020	12	16.8 y	Cholelithiasis	Laparoscopic cholecystectomy	ICG 0.4 mg/kg iv	Karl Storz + da Vinci Firefly, Intuitive	Retrospective study	2a
Esposito et al. [8]	2019	15	nd	Cholelithiasis	Laparoscopic cholecystectomy	ICG 0.4 mg/kg iv	Karl Storz	Retrospective study	2a
Esposito et al. [4]	2019	5	15.8 y	Cholelithiasis	Laparoscopic cholecystectomy	ICG 0.4 mg/kg iv	Karl Storz	Retrospective study	2a
Fernandez Bautista et al. [9]	2019	1	8.6 y	Cholelithiasis	Laparoscopic cholecystectomy	ICG 0.2 mg/kg iv	Stryker	Case series	1
Bryant et al. [6]	2020	1	17 y	Gall bladder duplication	Laparoscopic cholecystectomy	nd	nd	Case report	1
Calabro et al. [7]	2020	31	15 y	Symptomatic biliary diseases	Laparoscopic cholecystectomy	ICG 2.5 mg iv	Stryker	Prospective study	2a
Yanagi et al. [11]	2019	10	74.8 d	Biliary atresia	Kasai portoenterostomy (*n* = 9); hepaticojejunostomy (*n* = 1)	ICG 0.5 mg/kg iv	Karl Storz	Retrospective study	2a
Hirayama et al. [12]	2015	5	52 d	Biliary atresia	Kasai portoenterostomy	ICG 0.1 mg/kg iv	Hamamatsu	Retrospective study	2a
Hanaki et al. [13]	2022	1	nd	Biliary leakage after hepatectomy	Suture repair of biliary leak	ICG 2 mg iv	Stryker	Case report	1

Abbrev. d: days; y: years; iv: intravenously; pt: patient, nd: no data.

#### 3.1.2. Vascular Perfusion for Gastrointestinal (GI) Procedures 

Our search yielded 8 articles regarding vascular perfusion applied for GI procedures. Six of them were case reports, one was a retrospective study, and one was a prospective study. In total, 28 cases were reported, with 13 being the maximum number of patients reported in any study. (Table 2).

A total of 5 articles described fluorescent angiography to assess bowel perfusion: 8 bowel resections and stoma creations for necrotizing enterocolitis [14], 1 PSARP [15], 9 PSARVUP, 4 pull-through [16], 1 primary intestinal anastomosis in small bowel volvulus [17], 2 colostomy closures for anorectal malformations (ARM) [18]. ICG fluorescence imaging was used to identify and manage insufficient graft perfusion in a boy who underwent liver transplantation [19]. In one case, fluorescence helped to detect the blood supply during a re-do Nissen [20] and, in another case, it was useful to validate blood perfusion of the esophageal wall and anastomotic site during esophageal atresia repair [21].

ICG was the dye employed in 7 out of 8 studies: it was injected intravenously (iv) intraoperatively in all 7 cases. Different doses were administered for each application. An amount of 0.2 mg/kg of ICG was injected during PSARP (*n* = 1), PSARVUP (*n* = 9), and pull-through (*n* = 4) [15,16]. The dye was injected iv in all procedures. There were no intraoperative adverse events during surgery, or any side effects related to the injection of the dye. ICG signal was detected in 100% of the cases. In particular, Rentea et al. [16] reported four patients with Cloaca and Hirschsprung’s disease where the bowel seemed well-perfused before ICG injection, but the fluorescence showed a hypoperfused bowel which was therefore resected. 

An amount of 25 mg of ICG diluted with distilled water to a concentration of 2.5 mg/mL was injected into a peripheral vein just before surgery in a 15-year-old boy who developed delayed intestinal stricture after undergoing massive intestinal resection due to a small intestinal volvulus [17]. No side effects were reported. ICG fluorescence angiography showed abnormal vascular flow patterns in the distal part of the jejunum, despite the apparent improvement of the serosal surface color. A retrospective quantitative analysis of the fluorescence intensity was conducted using a software program: changes in the fluorescence intensity over time were noted as the fluorescence intensity at the mesentery gradually increased over time; however, that observed at the distal jejunum demonstrated the absence of gradual increases over time. The authors of this case suggested that the measurement of intestinal perfusion based on conventional clinical assessment does not necessarily reflect the precise perfusion status in the intestine as intraoperative ICG does.

An amount of 0.3 mg/kg of ICG was administered during colostomy closure (*n* = 2) in patients with ARM [18]. The dye was intravenously injected after dissection of the colonic mesentery. Effective fluorescence was visualized within 30 s from injection in both cases. No adverse effects were reported. The authors suggested that the ICG fluorescence system was useful for pediatric colostomy closure to evaluate the intestinal perfusion before anastomosis and to evaluate the postoperative bowel function by detecting endoluminal ICG passage with the stool.

Intraoperative administration of 0.05 mg/kg of ICG was used to assess perfusion of the liver graft [19]. No side effects were shown.

A total of 0.125 mg/kg of ICG was infused to achieve visualization of the operative field in the case of re-do Nissen [20]. The dye was intravenously administered during surgery. Paraboschi et al. [20] suggested that the use of FGS significantly impacted intraoperative decision making as ICG helped identify and spare left gastric artery and intrabdominal esophagus blood supply and establish an adequate length of intra-abdominal esophagus. No postoperative complications occurred.

A total of 0.5 mg/kg of ICG was injected from a peripheral vein in a case of esophageal atresia to validate intraoperative blood perfusion of the esophageal wall and anastomotic site [21]. The fluorescence signal was easily recognized, and no side effects were reported.

Lastly, the only study that did not involve the use of ICG was that of Numanoglu et al. who employed fluorescein sodium (14 mg/kg injected during surgery) to detect bowel perfusion in patients affected by necrotizing enterocolitis (*n* = 8). The fluorescence signal was detected in 100% of cases, in particular, the fluorescein allowed the recognition of ischemic bowel segments in three patients which were not visible to the naked eye [14]. No side effects were reported.

Overall, FGS has proved to be effective in this field, both to detect blood perfusion during complex reconstructive procedures such as pull-throughs in cloacal reconstructions, Hirschsprung disease, PSARP, and re-do Nissen as well as to provide objective data about abnormal vascular flow patterns in cases of intestinal ischemia. However, prospective cohort studies need to be conducted to assess the advantages against the current standard technique. The nature of current studies places the FGS for this field in IDEAL Stage 1.

**Table 2:** Summary for vascular perfusion for gastrointestinal procedures.

Authors	Year	No. of Procedures Performed	Mean pt Age	Indication	Surgical Procedure	Dose of Dye	Imaging System	Study Design	IDEAL Framework Stage
Paraboschi et al. [15]	2022	1	6 m	ARM	PSARP	ICG 0.2 mg/kg iv	Medtronic	Case report	1
Yada et al. [18]	2020	3	13.5 m	ARM	Colostomy closure	ICG 0.3 mg/kg iv	Stryker	Case series	1
Rentea et al. [16]	2019	13	1.9 y	ARM, cloaca, Hirschsprung disease	PSARVUP (*n* = 9), primary and redo pull-through (*n* = 4)	ICG (range 0.1–0-3 mg/kg) iv	Stryker	Retrospective study	2a
Paraboschi et al. [20]	2022	1	17 y	GERD	Re-do Nissen	ICG 0.125 mg/kg iv	Medtronic	Case report	1
Onishi et al. [21]	2022	1	16 d	Esophageal atresia	Thoracoscopic anastomosis	ICG 0.5 mg/kg iv	Stryker	Case report	1
Kisaoglu et al. [19]	2019	1	4 y	Maple Syrup Urine Disease	Liver resection after transplantation	ICG 0.05 mg/kg iv	nd	Case report	1
Iinuma et al. [17]	2013	1	15 y	Intestinal volvulus	Primary intestinal anastomosis	ICG 25 mg iv	Hamamatsu	Case report	1
Numanoglu et al. [14]	2011	8	24.5 d	NEC	Laparoscopic bowel resection and stoma formation	Fluorescein 14 mg/kg iv	Karl Storz	Prospective study	2a

Abbrev. d: days; m: months; y: years; iv: intravenously; pt: patient; ARM: anorectal malformation; PSARP: posterior sagittal anorectoplasty; GERD: gastroesophageal reflux disease; NEC: necrotizing enterocolitis.

#### 3.1.3. Lymphatic Flow Imaging 

Our search yielded 12 articles regarding ICG fluorescent lymphography. Nine were case reports and three were retrospective studies. No multicenter study or RCT had been published yet. Overall, 33 cases were reported in this field, and the maximum number of patients reported in any study was 11. (Table 3).

In total, 6 authors reported 16 cases of FGS applied to identify iatrogenic vascular lesions or sites of primary leak in cases of chylothorax and chylous ascites [22,23,24,25,26,27]. ICG was the dye employed in each study; however, there is no consistency in the timing, dose, or site of injection. Some authors suggested injecting the ICG inter-toe one hour prior to surgery [22], others suggested multiple injections every 20 min, the first one into the dorsum of the left foot, the second one into the dorsum of the right food, and the third one into the dorsum of the left hand [23]. Moreover, Chang et al. [24] performed intraoperative fluorescence lymphography by injecting ICG subcutaneously at the bilateral inguinal region. Only in two cases was ICG not able to identify the site of the lymphatic leak [23,25]. In the remaining cases, fluorescence lymphatic imaging allowed the successful visualization of abnormal lymphatic drainage both in cases of postoperative chylous leakage and in cases of primary chylous ascites [22,23,24,26,27].

FGS was also employed in patients diagnosed with lymphatic malformations (LM) and lymphedema: 3 authors reported 14 cases of lymphedema where ICG was employed to guide lymphovenous anastomoses [28,29,30] and 3 articles showed 3 cases of ICG lymphography guiding resection of LM [31,32,33]. There is no clear indication about the dose and timing of the administration of ICG. Drobot et al. [32] reported a case of ICG lymphography in a 14-year-old girl suffering from an axillary macrocystic LM. Just before surgery, between 0.2 mL and 0.3 mL of 2.5 mg/mL of ICG solution was injected subcutaneously between the second and third digits and between the third and fourth digits of the left hand. Lymphography showed two functional lymphatic vessels that reached the cystic lesion margin, although LM itself did not have any fluorescent signal. Therefore, surgeons were able to perform a complete excision of the LM while avoiding unnecessary dissection of unconnected lymphatic vessels. Furthermore, postoperative lymphography confirmed the preservation of functional lymphatics and ruled out any damage or lymphatic leak.

Kato et al. [33] showed a case of a 11-month-old girl diagnosed with a large LM on her face. Through 12 injections of 0.02 mL of 0.25 mg/mL of ICG solution around the peri-orbital lymphangioma and observations with an infrared microscope, the authors were able to detect the exact location of lymphatic vessels and to reach the target lymphatic vessels with a small skin incision.

Shirota et al. [31] reported a case of abdominal LM resected using subcutaneous and intradermal injection of 0.05 mL (0.125 mg) of ICG 20 h prior to surgery. The fluorescence signal provided a real-time image of the LM, allowing the complete resection of the lesion. No side effects were reported in any cases. 

Indubitably, fluorescence lymphography can potentially be a precise tool for the identification of a chylous leakage site and lymphatic vessels; however, recommendations about the dose of dye, timing, and sites of injection need to be defined to demonstrate the benefits of fluorescent lymphography in pediatric surgery. The nature of the current studies in this field suggest that the evidence base is still at IDEAL Stage 1.

**Table 3:** Summary for lymphatic flow imaging.

Authors	Year	No. of Procedures Performed	Mean pt Age	Indication	Surgical Procedure	Dose of Dye	Imaging System	Study Design	IDEAL Framework Stage
Yokoyama et al. [27]	2020	1	2.5 m	Chylous ascites	Repairing of leak fibrin sealant with polyglycolic acid felt	ICG 0.1 mL id	Hamamatsu Photonics	Case report	1
Otake et al. [26]	2015	1	13 y	Chylous ascites	Laparoscopic ligation of the leakage site	nd	nd	Case report	1
Pham et al. [25]	2020	1	6 m	Postoperative chylothorax	Transcatheter occlusion of the thoracic duct	ICG 0.05 mL of 0.25 mg/mL id	nd	Case report	1
Shirotsuki et al. [22]	2018	11	range 1–25 d	Esophageal atresia + postoperative chylothorax	First thoracoscopic TEF repair (*n* = 8) and thoracoscopic repair of chylous leakage points (*n* = 3)	ICG 0.025 mg inter-toe injection	Karl Storz	Retrospective study	2a
Tan et al. [23]	2014	1	1 m	Postoperative chylothorax	Bilateral pleurodesis	ICG (id, 1st inj. 25 mcg, 2nd inj. 12.5 mcg, 3rd inj. 12.5 mcg)	nd	Case report	1
Chang et al. [24]	2014	1	3 m	Postoperative chylothorax	Open ligation of fistula	ICG 2 mL sc	nd	Case report	1
Cheng et al. [30]	2020	8	9.2 y	Primary lymphedema	Lymphovenous anastomosis or vascularized lymph node transfer	ICG 0.5 mL sd	Sony Corporation	Retrospective study	2a
Mihara et al. [29]	2015	5	15.5 m	Generalized lymphatic dysplasia	Lymphovenous anastomosis	ICG 0.1 mL sc	Hamamatsu Photonics	Retrospective study	2a
Ogata et al. [28]	2007	1	12 y	Lymphedema of the lower extremities	Lymphaticovenular anastomosis	ICG 0.2 mL id	Hamamatsu Photonics	Case report	1
Drobot et al. [32]	2020	1	14 y	Lymphatic malformation	Surgical excision	ICG 0.3 to 0.4 mL sc	Stryker	Case report	1
Kato et al. [33]	2017	1	11 m	Lymphatic malformation	Lymphatic venous anastomosis	ICG 0.02 mL sc	Zeiss	Case report	1
Shirota et al. [31]	2017	1	15 y	Lymphatic malformation	Surgical excision	ICG 0.125 mg sc and id	Hamamatsu Photonics	Case report	1

Abbrev. d: days; m: months; y: years; id: intradermal; pt: patient; sd: subdermally; sc: subcutaneously; TEF: tracheoesophageal fistula; inj: injection.

#### 3.1.4. Tumor Resection 

Our search yielded 20 eligible studies focusing on fluorescence imaging during benign and malignant tumor resections. Out of these 20 articles, 8 were case reports, 11 were retrospective studies, and 1 was a multicenter study involving 2 tertiary lever referral centers [34]. Each study described less than 100 procedures. In total, 238 cases were reported, with the maximum number of procedures performed in any study being 65 in 55 patients. (Table 4).

FGS was applied to identify a broad range of pediatric tumors, to visualize not only the primary tumor, the resection margins, and metastatic disease, but also key vasculature and lymphatic drainage.

In total, 10 out of 20 articles explored the use of fluorescence for liver resection and metastasectomies in cases of hepatoblastoma (HB). Overall, 136 fluorescence-guided procedures for HB have been published [35,36,37,38,39,40,41,42,43,44]. Intravenously injected ICG at a dose of 0.5 mg/kg was mainly used to identify the lesions. Regarding the timing of injection, the recommended interval between the injection of ICG and the surgery was approximately 72 h for primary liver HB. This is to allow greater washout of ICG from the adjacent normal liver tissue and increase the tumor-to-background ratio. Some authors advocate that longer intervals may reduce the sensitivity for detection of smaller lesions [37]. When resecting pulmonary metastases or other lesions, ICG was injected approximately 24 h before the surgery since there is no concern about background fluorescence. In all the studies mentioned, fluorescent imaging was able to provide clear visualization of the HB lesions or metastases [35,36,37,38,39,40,41,42,43,44]. Additionally, Cho et al. stated that in one case of their series, ICG was also able to reveal a nodule that was not detected during the preoperative scan which was subsequently pathologically diagnosed as HB [41]. Moreover, Shen et al. showed in their series that multiple small lesions, detected by intraoperative ICG fluorescence, were not previously demonstrable at the preoperative CT/MRI scans. These lesions were confirmed to be HB in the histopathology report [43]. Despite the clear accuracy of fluorescence in identifying HB lesions in certain cases, a high rate of false-positive lesions must also be taken into consideration: Kitagawa et al. indicated that among the 250 lung lesions resected, 29 specimens with fluorescent signal were actually pathologically negative. It is likely that the resected lesions were too small for histopathological detection or that tissues other than hepatoblastoma can radiate fluorescence. Further studies are needed to assess these hypotheses [39].

As for other tumors, 3 articles were published about fluorescence applications on Wilms tumor resection. Overall, 22 procedures were described [34,45,46]. ICG (1.5 mg/kg) was intravenously administered 24 h before surgery, with the aim of distinguishing between the tumor and the healthy kidney tissue similar to what was reported for liver tumors [45]. Interestingly, Abdelhafeez et al. demonstrated that Wilms tumors are hypo-fluorescent compared to healthy kidney tissue. Nevertheless, distinguishing renal tumors from surrounding normal kidneys was feasible and helpful in cases of nephron-sparing surgery [45].

A case report also showed the benefits of ICG injection into the normal renal parenchyma near the tumor in order to identify and sample lymph nodes after nephroureterectomy [46].

Additionally, in a multicenter study, two tertiary-level referral centers independently began ICG fluorescence-guided nodal mapping: in one center they performed mapping with ipsilateral intra-parenchymal injection of ICG during minimally invasive tumor nephrectomy and in the other center they conducted mapping with peri–hilar injection during open tumor nephrectomy. Lymphatic mapping was successful in 88% of patients [34].

Moreover, Esposito et al. [8,10] showed the application of FGS in cases of ovarian mass resection, lymph node thoracoscopic biopsy, and abdominal lymphoma excision. For the latter application, ICG (0.5 mg/kg) was administered intraoperatively to obtain a real-time visualization of bowel perfusion, define the ideal level of resection from the mass, and confirm adequate perfusion in cases of anastomosis.

Additionally, several papers explored the application of fluorescence in non-neoplastic lesions: one case of lung lesion resection entailed granulomatous inflammation with focal necrosis [47], one case of laparoscopic resection of a pancreatic nodule entailed a case of focal congenital hyperinsulinism [48], and one case of laparoscopic partial splenectomy entailed a splenic cyst [49]. Additionally, there was one case of thoracoscopic cystic adenomatoid malformation resection and one case of thoracoscopic pulmonary extra-lobar sequestration resection [10].

To conclude, as the largest published study in this field [42], by using ICG a broad range of pediatric malignant tumors consistently exhibited fluorescent signal compared to background tissue. These tumors included liver tumors (HBs, hepatocellular carcinomas), neuroblastomas, rhabdomyosarcomas, non-rhabdomyosarcomas, osteosarcomas, Ewing sarcoma, chondroblastoma, germ cells tumors, solid pseudopapillary neoplasms of the pancreas, lymphoma, and myoepithelial carcinoma. The sensitivity and specificity of ICG in detecting intraoperatively malignant tumor tissue was 88 and 77%. Additionally, the use of ICG in this study resulted in the recognition of four (6%) malignant lesions which would not have been identified by the alternative standard of care.

Despite the variety of the published literature in this field and the consensus reached on the time and dose of ICG injection, the lack of larger multicenter studies and prospective clinical trials suggests that the evidence base is still at IDEAL Stage 2a.

**Table 4:** Summary for tumor resection.

Authors	Year	No. of Procedures Performed	Mean pt age	Indication	Surgical Procedure	Dose of Dye	Imaging System	Study Design	IDEAL Framework Stage
Abdelhafeez et al. [34]	2022	8	median 2.5 y	Wilms tumor	Nephroureterectomy with lymph node sampling, MIS (*n* = 4), open (*n* = 4)	ICG 5 mg intraparenchymal (*n* = 4) or peri-hilar (*n* = 4)	Karl Storz (*n* = 4) + Visionsense Corp (*n* = 4)	Multicenter study	2a
Abdelhafeez et al. [45]	2022	12	median 3 y	Bilateral Wilms tumor (*n* = 7) + epithelioid angiomyolipoma (*n* = 1)	Bilateral nephron-sparing surgery (*n* = 3) + radical nephrectomy (*n* = 1) + unilateral nephron-sparing surgery (*n* = 5)	ICG 1.5 mg/kg iv	Visionsense Corp	Retrospective study	2a
Pachl et al. [46]	2021	1	2 y	Wilms tumor	Laparoscopic nephroureterectomy + resection of nodes	2 mL of 2.5mg/mL ICG	Karl Storz	Case report	1
Delgado-Miguel et al. [48]	2022	1	3 m	Focal congenital hyperinsulinism	Laparoscopic resection of pancreatic nodule	ICG 2 mg/kg iv	Stryker	Case report	1
Chung et al. [24]	2020	1	9 y	Hepatocellular carcinoma	Laparoscopic segment 5th resection	ICG 0.5 mg/kg iv	nd	Case report	1
Bada-Bosch et al. [49]	2020	1	13 y	Splenic cyst	Laparoscopic partial splenectomy	ICG 0.2 mg/kg iv	Stryker	Case report	1
Mansfield et al. [50]	2020	4	15.7 y	Paratesticular RMS	Retroperitoneal lymph node dissection	nd	nd	Retrospective study	2a
Fung et al. [47]	2020	1	4 y	Lung lesion	Thoracoscopic resection of the lesion	Methylene blue (0.5 mL) and ICG (0.5 mL) inj. around the lesion via the guiding needle	Karl Storz	Case report	1
Esposito et al. [10]	2020	11	6.2 y	Abdominal lymphoma (*n* = 3) + ovarian tumors (*n* = 5) + CAM (*n* = 1) + pulmonary sequestration (*n* = 1) + lung hilar lymph node (*n* = 1)	Laparoscopic abdominal lymphoma excisions (*n* = 3) + robotic ovarian mass excision (*n* = 5) + thoracoscopic lobectomy (*n* = 2) + thoracoscopic biopsy (*n* = 1)	ICG 0.5 mg/mL/kg iv	Karl Storz + Da vinci Firefly, Intuitive	Retrospective study	2a
Esposito et al. [8]	2019	6	3.8 y	Abdominal lymphoma (*n* = 3) + abdominal tumor (*n* = 3)	Laparoscopic excision	ICG 0.5 mg/kg iv	Karl Storz	Retrospective study	2a
Shen et al. [43]	2022	16	median 15 m	HB	Liver resection	ICG 0.5 mg/kg iv	nd	Retrospective study	2a
Abdelhafeez et al. [42]	2021	65	median 10 y	Thoracic lesions (*n* = 37) + abdomen masses (*n* = 19) + lesions trunk and extremities (*n* = 9)	Excision of HB (*n* = 9) + HCC (*n* = 2) + OS (*n* = 9) + NB (*n* = 6) + NRSTS (*n* = 6) + RMS (*n* = 5) + ES (*n* = 3) + GCT (*n* = 2) + CB (*n* = 1) + SPNP (*n* = 1) + Lymphoma (*n* = 1) + myoepithelial carcinoma of the chest wall (*n* = 1) + ACT (*n* = 2) + metastasectomy (*n* = 4) + non tumor resection (*n* = 13)	ICG 1.5 mg/kg iv	Visionsense Corp	Retrospective study	2a
Cho et al. [41]	2021	22	3 y	HB	Liver resections (*n* = 17) + liver transplants (*n* = 2) + lung metastasectomy (*n* = 2) + lymph-node metastasis sampling (*n* = 1)	ICG 0.3 mg/kg iv	Karl Storz	Retrospective study	2a
Souzaki et al. [35]	2019	10	2.5 y	Primary liver tumors (*n* = 4), lung metastases (*n* = 6)	Extended right hepatectomy (*n* = 3) + liver transplantation (*n* = 1) + lung partial resection (*n* = 5) + lobectomy (*n* = 1)	ICG 0.5 mg/kg iv	Karl Storz	Retrospective study	2a
Takahashi et al. [36]	2019	1	14 y	HB peritoneal dissemination	Surgical excision + Living Donor Liver ReTransplantation	ICG 0.5 mg/kg iv	Hamamatsu Photonics	Case report	1
Yamada et al. [37]	2019	36	5 y	Primary HB (*n* = 12), HB lung metastases (*n* = 7), mediastinal metastasis (*n* = 1), peritoneal metastasis (*n* = 1), pancreatic metastasis (*n* = 1), bone metastasis (*n* = 1)	Liver resection (*n* = 13), lung metastasectomies (*n* = 15), other metastasectomies (*n* = 5)	ICG 0.5 mg/kg iv	Hamamatsu Photonics	Retrospective study	2a
Chen-Yoshikawa et al. [38]	2017	1	3 y	HB lung metastasis	Lung metastasectomy	ICG 0.5 mg/kg iv	Panasonic AVC Networks Company	Case report	1
Kitagawa et al. [39]	2015	37	3.5 y	HB lung metastases (*n* = 10)	Lung metastasectomy (*n* = 37)	ICG 0.5 mg/kg iv	Hamamatsu Photonics	Retrospective study	2a
Yamamichi et al. [40]	2015	3	3 y	Primary HB (*n* = 1), recurrent HB (*n* = 1), HB lung metastasis (*n* = 1)	Right hepatectomy (*n* = 1), residual tumor and diaphragm resection (*n* = 1), lung metastasectomy (*n* = 1)	ICG 0.5 mg/kg iv	Mizuho Medical Co	Case series	1
Mitani et al. [44]	2014	1	2.6 y	HB	Tumor resection	ICG 0.5 mg/kg iv	Hamamatsu Photonics	Case report	1

Abbrev. m: months; y: years; MIS: minimally invasive surgery; iv: intravenously; inj: injected; pt: patient; OS: osteosarcoma; HB: hepatoblastoma; ES: Ewing sarcoma; RMS: rhabdomyosarcoma; NRSTS: non-rhabdomyosarcoma soft tissue sarcoma; CB: chondroblastoma; NB: neuroblastoma; HCC: hepatocellular carcinoma; GCT: germ cell tumor, SPNP: solid pseudopapillary neoplasm of the pancreas; ACT: adrenocortical tumor, CAM: cystic adenomatoid malformation.

#### 3.1.5. Urogenital Surgery 

Our search yielded 9 articles focusing on FGS employed during urogenital procedures. All 9 articles were retrospective studies, involving less than 100 patients each. In total, 197 cases were reported, the maximum number of procedures performed in any study being 57. (Table 5).

The main applications were the detection of blood and lymphatic vessels during varicocelectomies (*n* = 137) [8,9,10,51,52], the visualization of hilar vessels and kidney perfusion during heminephrectomies (*n* = 36) [8,10,52,53,54], nephrectomies (*n* = 11) [8,9,10,52], renal cysts deroofing (*n* = 10) [10,52,55], and the detection of ureteral perfusion after ureteral reconstruction (*n* = 3) [56].

During laparoscopic varicocelectomies, according to Esposito et al., 2 mL of ICG solution (25 mg of ICG with 8ml of sterile water) was intraoperatively injected into the testis to detect and spare the lymphatics avoiding the risk of postoperative hydrocele. Under near-infrared light, the lymphatics were identified, appearing fluorescent. No adverse reactions were reported [8,10,51,52]. In one study, Fernández-Bautista et al. administrated ICG intravenously at a dose of 0.2 mg/kg, showing good results and no side effects [9].

During heminephrectomies, according to Esposito et al., ICG was injected into a peripheral vein at a dosage of 0.3 to 0.5 mg/kg just after the dissection of Gerota’s fascia. No adverse effects were reported. According to Herz et al., 0.5–1.0 mL of ICG solution (2.5 mg/mL), administered 30–60 s prior to surgery also showed good results and no side effects. Herz et al. described the advantages of arterial mapping during pediatric heminephrectomies attesting that ICG imaging helped detect the correct area of excision and in one case helped avoid a potential critical complication by identifying an artery to the healthy moiety [53]. In 2021 Esposito et al. [54] standardized the technique of ICG injection for partial nephrectomy in three different steps: the first injection was performed just prior to surgery into the ureteral catheter to identify the ureter. Then, ICG was injected intravenously to identify the hilar vessel and the vasculature of the non-functioning portion. After ligation of the vessels supplying the non-functioning renal part, the third injection was performed intravenously to identify the boundary plane between the avascular and the perfused pole and correctly guide the resection of the parenchyma. In this study, Esposito et al. showed that in the group of ICG-guided laparoscopic nephrectomies no intraoperative complication occurred, and the incidence of postoperative renal cysts was also significantly lower compared with the standard technique.

Considering nephrectomies, Esposito et al. [10,52] showed that ICG (dosage 0.3 mg/mL/kg) was injected intravenously just after the division of the Gerota’s fascia and the fluorescence signal was visualized in the hilar renal vessels in less than 2 min and in the renal parenchyma within a few seconds after the renal vessels. No intra or postoperative complications occurred. In another study, Esposito et al. [8] showed that ICG was injected using a dosage of 0.5 mg/kg. with the same good results and no complications or side effects. In one study, Fernández-Bautista et al. administrated ICG intravenously at a dose of 0.2 mg/kg with good results [9].

During renal cyst deroofing, ICG (dosage 0.3 mg/mL/kg) was injected intraoperatively and intravenously just after the division of the Gerota’s fascia. In just one minute the fluorescence allowed the “non-fluorescent” area corresponding to the avascular cyst to be clearly distinguished from the “fluorescent green” area corresponding to the perfused renal parenchyma. No complications or side effects occurred [10,52,55].

Finally, Carty et al. [56] showed that ICG injected intravenously at a dosage of 0.039–0.086 mg/kg was used to elucidate blood supply to the affected segment of the ureter within 60 s of administration in cases of ureteral reconstruction.

Overall, the modality, timing, and dosage of ICG administration seem to be standardized for each procedure in this category; however, the main limitation is that only single centers’ experiences have been reported. Multicenter studies and prospective clinical trials need to be performed to demonstrate the clear advantages of FGS for such indications. The evidence base for this field is still at IDEAL Stage 2a.

**Table 5:** Summary for urogenital surgery.

Authors	Year	No. of Procedures Performed	Mean pt Age	Indication	Surgical Procedure	Dose of Dye	Imaging System	Study Design	IDEAL Framework Stage
Esposito et al. [54]	2021	12	median 4.1 y	Duplex kidney	Laparoscopic partial nephrectomy	ICG 0.3 mg/kg iv or into the ureteral catheter	Karl Storz	Retrospective study	2a
Herz et al. [53]	2016	6	5.6 y	Duplex kidney	Robot-assisted laparoscopic heminephrectomy	ICG 1.25–2.5 mg iv	Da Vinci Firefly, Intuitive	Retrospective study	2a
Carty et al. [56]	2021	3	8 y	Congenital ureteral stricture, mid-ureteral polyp disease, distal-ureteral polyp disease	Ureteral reconstruction	ICG 0.039–0.086 mg/kg iv	Da Vinci Firefly, Intuitive	Case series	1
Esposito et al. [55]	2020	3	nd	Solitary renal cyst	Robotic deroofing of cyst	ICG 0.35mg/kg iv	nd	Retrospective study	1
Esposito et al. [52]	2020	57	median 15.7 y (varicocele) + 8.7 y (nephrectomy) + 4.3 y (partial nephrectomy) + 10.8 y (renal cyst)	Varicocele (*n* = 41) + non-functioning kidney (*n* = 3) + symptomatic non-functioning obstructive upper pole moiety or lower pole moiety (*n* = 9) + simple renal cyst (*n* = 4)	Laparoscopic left varicocelectomy (*n* = 38) + robot-assisted left varicocelectomy (*n* = 3) + nephrectomy (*n* = 3) + partial nephrectomy (*n* = 9) + robot-assisted deroofing of simple renal cyst (*n* = 4)	ICG 0.3 mg/mL/kg iv	Karl Storz + Da Vinci Firefly, Intuitive	Retrospective study	2a
Esposito et al. [10]	2020	53	10.9 y	Varicocele (*n* = 40) + non-functioning kidney (*n* = 3) + non-functioning symptomatic obstructive upper pole or lower pole moiety (*n* = 7) + + simple renal cyst (*n* = 3)	Left varicocelectomy (*n* = 40), partial nephrectomy (*n* = 7), nephrectomy (*n* = 3), renal cyst deroofing (*n* = 3)	ICG 6.25 mg intratesticular (varicocele repair) + 0.3 mg/mL/kg iv (nephrectomy and renal cyst deroofing)	Karl Storz + Da Vinci Firefly, Intuitive	Retrospective study	2a
Esposito et al. [51]	2019	25	13.7 y	Varicocele	Laparoscopic Palomo left varicocelectomy	ICG 0.1 mg intratesticular	nd	Retrospective study	2a
Esposito et al. [50]	2019	35	8.4 y	Varicocele (*n* = 30) + non-functioning kidney (*n* = 3) + non-functioning upper pole in duplex kidney (*n* = 2)	Laparoscopic Palomo left varicocelectomy (*n* = 30) + nephrectomy (*n* = 3) + partial nephrectomy (*n* = 2)	ICG 2 mL intratesticular + 0.5 mg/kg iv (renal surgery)	Karl Storz	Retrospective study	2a
Fernandez Bautista et al. [9]	2019	3	8.6 y	Varicocele (*n* = 1) + renal failure (*n* = 2)	Laparoscopic Palomo varicocelectomy (*n* = 1) + laparoscopic nephrectomy (*n* = 2)	ICG 0.2 mg/kg iv	Stryker	Case series	1

Abbr. y: years; iv: intravenously.

#### 3.1.6. Plastic Surgery 

Our search yielded four articles regarding fluorescent imaging applications in plastic surgery. Two of them were case reports and two were retrospective studies. In total, 26 cases were reported with the maximum number of patients reported in any study being 13. (Table 6).

The main applications were the detection of flap perfusion, the assessment of vessels patency during revascularization procedures, and the visualization of vessels’ course and flow.

In this field, intraoperative ICG angiography has the potential to be an objective method to assess intraoperative flap perfusion, thus enabling the surgeon to take additional measures to avoid any ischemic problems.

Martins et al. [57] showed how to assess skin flap perfusion for pediatric autologous ear reconstruction using relative and absolute perfusion units (RPU and APU) color maps generated intraoperatively by the SPY-Q software. In particular, they suggested that in cases of skin flap necrosis, the lowest APU was < 20. However, low APU values need to be clinically related to ascertain that the irregularity of the tissue is not responsible for the decreased fluorescent signal. Their study concluded showing a decreased number of surgical revisions in cases treated with ICG injection versus those without ICG, suggesting that greater certainty in skin flap perfusion is related to a reduction in revision surgeries [57].

Fluorescence imaging was also applied during percutaneous sclerotherapy of venous malformations, especially in the face and hands, allowing non-invasive assessment of the real-time distribution of sclerosant [58].

Only a few studies have been published on this technique, therefore, more cases and multicenter studies are needed to demonstrate the benefits of ICG fluorescence imaging in plastic surgery. The nature of the current studies for this field places the FGS in IDEAL Stage 1/2a.

**Table 6:** Summary for plastic surgery.

Authors	Year	No. of Procedures Performed	Mean pt Age	Indication	Surgical Procedure	Dose of Dye	Imaging System	Study Design	IDEAL Framework Stage
Fried et al. [59]	2019	1	6 m	Teratoma	Free latissimum dorsi myocutaneous flap	nd	nd	Case report	1
Martins et al. [57]	2016	11	8.8 y	nd	First-stage autologous rib cartilage ear reconstructions	ICG 5 mg iv	Stryker	Retrospective study	2a
Hinchcliff et al. [60]	2013	1	1 y	Anterior plagiocephaly	Reconstruction for left unilateral coronal synostosis	ICG 2.5 mg iv	Stryker	Case report	1
Ishikawa et al. [58]	2013	13	10.5 y	Venous malformations	Sclerotherapy	0.04 mL of ICG (0.01 mg/mL) injected inside the malformation	Hamamatsu Photonics	Retrospective study	2a

Abbr. m: months; y: years; iv: intravenously; pt: patients.

#### 3.1.7. Miscellaneous 

Only one case report could not be assigned to any of the previously described main groups: it reported a case of a 13-day-old patient who underwent ICG administration into the stomach during a laparoscopic duodenal web excision [61]. (Table 7). Just after anesthesia, 5 mL of ICG solution (2.5 mg/mL) was injected via the nasogastric tube, then the location of the duodenal web was accurately identified in real-time. After the excision of the duodenal web, another dose of 5 mL was injected to confirm the patency of the duodeno-duodenostomy.

Determining the exact location of a duodenal web can be challenging, especially during minimally invasive surgery or if the web has a wind-sock shape. In this case report, fluorescence allowed the surgeon to identify the exact location of the web and guided the location of the proximal and distal duodenotomies. No intraoperative complications or side effects occurred.

This application could be expanded for each intestinal atresia to assess the patency of the bowel and exclude multiple areas of atresia.

A limitation of this study was that as this procedure was only used for one patient, more research is needed to fully understand the benefits.

## 4. Discussion

This systematic review is the first study publishing data about the development status of FGS in pediatric surgery using the IDEAL framework. Our aim was not only to identify the state of research of FGS in pediatrics but also to suggest further studies to be performed in the future, to mature this novel surgical technique into a standardized and effective technique.

Since the last systematic review [62], the number of cases published has tripled, showing that interest in FGS in pediatric surgery has been increasing steadily and widely in the last few years. However, there is still a lack of robust evidence supporting its use in the pediatric population.

The most important limit is the nature of the studies published, which are mostly small case series and single-center studies. Moreover, for some applications, there is an extraordinary heterogeneity across the studies: indications, ICG dose, and outcomes of FGS are extremely variable. A uniform consensus on the dose and timing of ICG injection is not reached even in similar indications.

Besides this, the level of evidence varies across the different surgical fields. For example, fluorescence imaging appears mostly beneficial for delineating hepatobiliary structures and vascular perfusion in urogenital surgery and tumor resection. Indeed, our IDEAL framework analysis indicates that the evidence base is in Stage 2a (Development) for these three fields, as the technique is considered safe and has been standardized. Prospective and collaborative cohort studies are now appropriate to finalize the consensus on methods and outcome measures for a future RCT.

In the oncological field, FGS has been developing as a cutting-edge improvement in which precise margin definitions have the potential to lead to more radical tumor resections and better outcomes. The recent increase in publications in this field makes the tumor resection category almost ready to proceed to IDEAL stage 2b. Prospective studies to assess the effectiveness of the new technique against current standards will be pivotal to reaching this next stage.

To reach the next IDEAL stage, it is not only important to accumulate more cases, but it is also important to achieve a more uniform protocol for establishing the optimal timing and dosage for ICG injection and for a more defined selection of patients with regard to tumor depth from the surface. In fact, as Cho et al. stated, tumors at a depth of <10 mm in the liver/lung surface were all visible under the ICG camera but one of the clear current limitations for its clinical use is related to the limited tissue penetration (up to 10 mm) of near-infrared light [42,60].

On the other hand, some fields of pediatric surgery are still in the early phase of adoption. For example, fluorescence imaging for vascular perfusion in GI procedures and lymphatic flow imaging are at an IDEAL stage 1. The reason for this is that most articles report single-center experiences for a small number of patients involved. To make more broad conclusions about the effectiveness of FGS, more studies need to be published to assess its effectiveness in these fields of pediatrics.

Although the literature supports the useful role of ICG imaging in the evaluation of flaps perfusion, there seems to be less interest in employing fluorescence imaging in plastic surgery considering that only four reports emerged from our research. An explanation could be that, despite the clear benefits of fluorescence imaging, equipment and ICG access and time constraints in the operating theatre may limit its daily adoption.

Despite the recent advances in FGS, further developments are still feasible. In this regard, in the field of pediatric cancer surgery, preclinical studies are investigating new technologies to maximize the fluorescent signal from the tumor and to minimize the background noise. NIR-II (wavelength: 1000–2000 nm) fluorophores and tumor-targeted fluorescent probes seem to be promising tools for achieving higher contrast, increased sensitivity, and improved tissue penetration depths [63,64].

Finally, complementary analytical tools, such as artificial intelligence (AI), will be integrated into an FGS system to enhance the decision-making capability of fluorescence optical imaging. In this aspect, one of the most revolutionizing breakthroughs was the introduction of the Internet of things (IoT) concept within surgical practice. Internet-based image-guided surgery has a lot to offer by incorporating data from different sources and making them available to the surgeon in real-time, for example by allowing the surgeon to visualize preoperative and intraoperative images of the anatomical structures of interest overlaid in real-time [65].

## 5. Conclusions

FGS applied to biliary tree imaging, urogenital surgery, and tumor resection is safe and feasible in pediatric surgery, providing real-time information for surgical decision-making. Multicenter, prospective clinical studies, and RCT are required to standardize the use of FGS in these fields of application.

Lymphatic flow evaluation, vascular assessment for GI procedures, and plastic surgery are other promising applications of fluorescent optical imaging in children, but more cases need to be published to evaluate their effectiveness in pediatrics.

## Figures and Tables

**Table 7:** The only case not belonging to any of the other groups.

Authors	Year	No. of Procedures Performed	Mean pt Age	Indication	Surgical Procedure	Dose of Dye	Imaging System	Study Design	IDEAL Framework Stage
Li S et al. [61]	2022	1	13 d	Duodenal web	Laparoscopic resection of duodenal web	ICG 5 mL of a 0.125 mg/mL diluted solution through a nasogastric tube	Zhuhai Dipu Medical Technology Co	Zhuhai Dipu Medical Technology Co	1

Abbr. d: days; pt: patients.

## Data Availability

Data sharing not applicable.

## References

[1] Ishizawa T., McCulloch P., Muehrcke D., Carus T., Wiesel O., Dapri G., Schneider-Koriath S., Wexner S.D., Abu-Gazala M., Boni L. (2021). Assessing the development status of intraoperative fluorescence imaging for perfusion assessments, using the IDEAL framework. BMJ Surg. Interv. Health Technol..

[2] McCulloch P., Altman D.G., Campbell W.B., Flum D.R., Glasziou P., Marshall J.C., Nicholl J. (2009). No surgical innovation without evaluation: The IDEAL recommendations. Lancet.

[3] Pennell C.P., Hirst A.D., Campbell W.B., Sood A., Agha R.A., Barkun J.S.T., McCulloch P. (2016). Practical guide to the Idea, Development and Exploration stages of the IDEAL Framework and Recommendations. Br. J. Surg..

[4] Esposito C., Corcione F., Settimi A., Farina A., Centonze A., Esposito G., Spagnuolo M.I., Escolino M. (2019). Twenty-Five Year Experience with Laparoscopic Cholecystectomy in the Pediatric Population—From 10 mm Clips to Indocyanine Green Fluorescence Technology: Long-Term Results and Technical Considerations. J. Laparoendosc. Adv. Surg. Tech..

[5] Esposito C., Settimi A., Cerulo M., Escolino M. (2022). Efficacy of indocyanine green (ICG) fluorescent cholangiography to improve intra-operative visualization during laparoscopic cholecystectomy in pediatric patients: A comparative study between ICG-guided fluorescence and standard technique. Surg. Endosc..

[6] Bryant M.K., Marulanda K., Phillips M.R. (2020). Laparoscopic Double Cholecystectomy in a Pediatric Patient for Gallbladder Duplication: An Unusual Case of Biliary Anatomy. Am. Surg..

[7] Calabro K.A., Harmon C.M., Vali K. (2020). Fluorescent Cholangiography in Laparoscopic Cholecystectomy and the Use in Pediatric Patients. J. Laparoendosc. Adv. Surg. Tech..

[8] Esposito C., Del Conte F., Cerulo M., Gargiulo F., Izzo S., Esposito G., Spagnuolo M.I., Escolino M. (2019). Clinical application and technical standardization of indocyanine green (ICG) fluorescence imaging in pediatric minimally invasive surgery. Pediatr. Surg. Int..

[9] Fernández-Bautista B., Mata D.P., Parente A., Pérez-Caballero R., De Agustín J.C. (2019). First Experience with Fluorescence in Pediatric Laparoscopy. Eur. J. Pediatr. Surg. Rep..

[10] Esposito C., Settimi A., Del Conte F., Cerulo M., Coppola V., Farina A., Crocetto F., Ricciardi E., Esposito G., Escolino M. (2020). Image-Guided Pediatric Surgery Using Indocyanine Green (ICG) Fluorescence in Laparoscopic and Robotic Surgery. Front. Pediatr..

[11] Yanagi Y., Yoshimaru K., Matsuura T., Shibui Y., Kohashi K., Takahashi Y., Obata S., Sozaki R., Izaki T., Taguchi T. (2019). The outcome of real-time evaluation of biliary flow using near-infrared fluorescence cholangiography with Indocyanine green in biliary atresia surgery. J. Pediatr. Surg..

[12] Hirayama Y., Iinuma Y., Yokoyama N., Otani T., Masui D., Komatsuzaki N., Higashidate N., Tsuruhisa S., Iida H., Nakaya K. (2015). Near-infrared fluorescence cholangiography with indocyanine green for biliary atresia. Real-time imaging during the Kasai procedure: A pilot study. Pediatr. Surg. Int..

[13] Hanaki T., Tokuyasu N., Sakamoto T., Fujiwara Y. (2022). Hepatectomy guided by indocyanine green fluorescent imaging for visualizing bile leakage (with video). Clin. Case Rep..

[14] Numanoglu A., Millar A.J.W. (2011). Necrotizing enterocolitis: Early conventional and fluorescein laparoscopic assessment. J. Pediatr. Surg..

[15] Paraboschi I., Privitera L., Loukogeorgakis S., Giuliani S. (2022). Indocyanine Green-Based Fluorescence-Guided Surgery in a Male Infant with Anorectal Malformation. Eur. J Pediatr. Surg. Rep..

[16] Rentea R.M., Halleran D.R., Ahmad H., Sanchez A.V., Gasior A.C., McCracken K., Hewitt G.D., Alexander V., Smith C., Weaver L. (2020). Preliminary Use of Indocyanine Green Fluorescence Angiography and Value in Predicting the Vascular Supply of Tissues Needed to Perform Cloacal, Anorectal Malformation, and Hirschsprung Reconstructions. Eur. J. Pediatr. Surg..

[17] Iinuma Y., Hirayama Y., Yokoyama N., Otani T., Nitta K., Hashidate H., Yoshida M., Iida H., Masui D., Manabe S. (2013). Intraoperative near-infrared indocyanine green fluorescence angiography (NIR-ICG AG) can predict delayed small bowel stricture after ischemic intestinal injury: Report of a case. J. Pediatr. Surg..

[18] Yada K., Migita M., Nakamura R., Abe S., Matsufuji H. (2020). Indocyanine green fluorescence during pediatric stoma closure. J. Pediatr. Surg. Case Rep..

[19] Kisaoglu A., Demiryilmaz I., Dandin O., Ozkan O., Aydinli B. (2020). Management of reperfusion deficiency with indocyanine green fluorescence imaging during deceased donor liver transplantation in a pediatric recipient. HPB.

[20] Paraboschi I., Privitera L., Loukogeorgakis S., Giuliani S. (2022). Fluorescence-Guided Surgery (FGS) during a Laparoscopic Redo Nissen Fundoplication: The First Case in Children. Children.

[21] Onishi S., Muto M., Yamada K., Murakami M., Kedoin C., Nagano A., Matsui M., Sugita K., Yano K., Harumatsu T. (2022). Feasibility of delayed anastomosis for long gap esophageal atresia in the neonatal period using internal traction and indocyanine green-guided near-infrared fluorescence. Asian J. Endosc. Surg..

[22] Shirotsuki R., Uchida H., Tanaka Y., Shirota C., Yokota K., Murase N., Hinoki A., Oshima K., Chiba K., Sumida W. (2018). Novel thoracoscopic navigation surgery for neonatal chylothorax using indocyanine-green fluorescent lymphography. J. Pediatr. Surg..

[23] Tan I.-C., Balaguru D., Rasmussen J.C., Guilliod R., Bricker J.T., Douglas W.I., Sevick-Muraca E.M. (2014). Investigational Lymphatic Imaging at the Bedside in a Pediatric Postoperative Chylothorax Patient. Pediatr. Cardiol..

[24] Chang T.-I., Chen Y.-S., Huang S.-C. (2014). Intraoperative indocyanine green fluorescence lymphography to detect chylous leakage sites after congenital heart surgery. J. Thorac. Cardiovasc. Surg..

[25] Pham K.T., Balaguru D., Tammisetti V.S., Guevara C.J., Rasmussen J.C., Zvavanjanja R.C., Hanfland R., Sevick-Muraca E.M., Aldrich M.B. (2020). Multimodality lymphatic imaging of postoperative chylothorax in an infant with Noonan syndrome: A case report. Eur. J. Med. Res..

[26] Otake K., Uchida K., Inoue M., Koike Y., Narushima M., Kusunoki M. (2015). Use of computed tomography-lymphangiography with direct injection of water-soluble contrast medium to identify the origin of chylous ascites. J. Vasc. Surg. Venous Lymphat. Disord..

[27] Yokoyama S., Nakaoka T. (2020). Successful use of intraoperative ICG fluorescence lymphography and fibrin sealant with PGA felt for refractory chylous ascites in an infant: A novel procedure. Pediatr. Int..

[28] Ogata F., Narushima M., Mihara M., Azuma R., Morimoto Y., Koshima I. (2007). Intraoperative Lymphography Using Indocyanine Green Dye for Near-Infrared Fluorescence Labeling in Lymphedema. Ann. Plast. Surg..

[29] Mihara M., Hara H., Shibasaki J., Seki Y., Hayashi A., Iida T., Adachi S., Uchida Y., Kaneko H., Haragi M. (2015). Indocyanine Green Lymphography and Lymphaticovenous Anastomosis for Generalized Lymphatic Dysplasia with Pleural Effusion and Ascites in Neonates. Ann. Vasc. Surg..

[30] Cheng M., Liu T.T. (2020). Lymphedema microsurgery improved outcomes of pediatric primary extremity lymphedema. Microsurgery.

[31] Shirota C., Hinoki A., Takahashi M., Tanaka Y., Tainaka T., Sumida W., Murase N., Oshima K., Shirotsuki R., Chiba K. (2017). New Navigation Surgery for Resection of Lymphatic Malformations Using Indocyanine Green Fluorescence Imaging. Am. J. Case Rep..

[32] Drobot A., Ganam S., Karra N., Bickel A., Abu Shakra I., Kakiashvili E. (2021). Resection of an axillary macrocystic lymphatic malformation in a 14-year-old girl using intraoperative indocyanine green lymphography. J. Vasc. Surg. Venous Lymphat. Disord..

[33] Kato M., Watanabe S., Iida T., Watanabe A., Megumi F. (2017). Peri-orbital lymphangioma treated by lymphatic-venous anastomosis with indocyanine green lymphography analysis. J. Pediatr. Surg. Case Rep..

[34] Abdelhafeez A.H., Davidoff A.M., Murphy A.J., Arul G.S., Pachl M.J. (2022). Fluorescence-guided lymph node sampling is feasible during up-front or delayed nephrectomy for Wilms tumor. J. Pediatr. Surg..

[35] Souzaki R., Kawakubo N., Matsuura T., Yoshimaru K., Koga Y., Takemoto J., Shibui Y., Kohashi K., Hayashida M., Oda Y. (2019). Navigation surgery using indocyanine green fluorescent imaging for hepatoblastoma patients. Pediatr. Surg. Int..

[36] Takahashi N., Yamada Y., Hoshino K., Kawaida M., Mori T., Abe K., Fujimura T., Matsubara K., Hibi T., Shinoda M. (2019). Living Donor Liver Re-Transplantation for Recurrent Hepatoblastoma in the Liver Graft following Complete Eradication of Peritoneal Metastases under Indocyanine Green Fluorescence Imaging. Cancers.

[37] Yamada Y., Ohno M., Fujino A., Kanamori Y., Irie R., Yoshioka T., Miyazaki O., Uchida H., Fukuda A., Sakamoto S. (2019). Fluorescence-Guided Surgery for Hepatoblastoma with Indocyanine Green. Cancers.

[38] Chen-Yoshikawa T.F., Hatano E., Yoshizawa A., Date H. (2017). Clinical application of projection mapping technology for surgical resection of lung metastasis. Interact. CardioVascular Thorac. Surg..

[39] Kitagawa N., Shinkai M., Mochizuki K., Usui H., Miyagi H., Nakamura K., Tanaka M., Tanaka Y., Kusano M., Ohtsubo S. (2015). Navigation using indocyanine green fluorescence imaging for hepatoblastoma pulmonary metastases surgery. Pediatr. Surg. Int..

[40] Yamamichi T., Oue T., Yonekura T., Owari M., Nakahata K., Umeda S., Nara K., Ueno T., Uehara S., Usui N. (2015). Clinical application of indocyanine green (ICG) fluorescent imaging of hepatoblastoma. J. Pediatr. Surg..

[41] Cho Y.J., Namgoong J.-M., Kwon H.H., Kwon Y.J., Kim D.Y., Kim S.C. (2021). The Advantages of Indocyanine Green Fluorescence Imaging in Detecting and Treating Pediatric Hepatoblastoma: A Preliminary Experience. Front. Pediatr..

[42] Abdelhafeez A., Talbot L., Murphy A.J., Davidoff A.M. (2021). Indocyanine Green–Guided Pediatric Tumor Resection: Approach, Utility, and Challenges. Front. Pediatr..

[43] Shen Y., Zheng M., Li J., Tan T., Yang J., Pan J., Hu C., Zou Y., Yang T. (2022). Clinical Application of Indocyanine Green Fluorescence Imaging in the Resection of Hepatoblastoma: A Single Institution’s Experiences. Front. Surg..

[44] Mitani Y., Kubota A., Ueno M., Takifuji K., Watanabe T., Hayami S., Kounami S., Tsujimoto H., Yamaue H. (2014). Real-time identification of hepatoblastoma using a near infrared imaging with indocyanine green. J. Pediatr. Surg. Case Rep..

[45] Abdelhafeez A.H., Murphy A.J., Brennan R., Santiago T.C., Lu Z., Krasin M.J., Bissler J.J., Gleason J.M., Davidoff A.M. (2022). Indocyanine green–guided nephron-sparing surgery for pediatric renal tumors. J. Pediatr. Surg..

[46] Pachl M.J. (2021). Fluorescent Guided Lymph Node Harvest in Laparoscopic Wilms Nephroureterectomy. Urology.

[47] Fung C., Lau C., Wong K.K. (2020). Indocyanine green fluorescence-guided pulmonary wedge resection in a child: A case report. Hong Kong Med. J..

[48] Delgado-Miguel C., Muñoz-Serrano A., Moratilla L., del Carmen Sarmiento M., Miguel-Ferrero M., Leal N., Barrena S., Martínez L. (2022). Indocyanine Green (ICG)-Guided Identification of Hypermetabolic Pancreatic Nodules in Focal Congenital Hyperinsulinism: A Case Report in a 3-Month-Old Infant. Eur. J. Pediatr. Surg. Rep..

[49] Bada-Bosch I., Mata D.P., de la Torre M., Ordóñez J., Blanco M.D., de Agustin J. (2020). Laparoscopic Partial Splenectomy Assisted by Fluorescence in a 13-Year-Old Girl. Eur. J. Pediatr. Surg. Rep..

[50] Mansfield S.A., Murphy A.J., Talbot L., Prajapati H., Maller V., Pappo A., Singhal S., Krasin M.J., Davidoff A.M., Abdelhafeez A. (2020). Alternative approaches to retroperitoneal lymph node dissection for paratesticular rhabdomyosarcoma. J. Pediatr. Surg..

[51] Esposito C., Turrà F., Del Conte F., Izzo S., Gargiulo F., Farina A., Severino G., Cerulo M., Escolino M. (2019). Indocyanine Green Fluorescence Lymphography: A New Technique to Perform Lymphatic Sparing Laparoscopic Palomo Varicocelectomy in Children. J. Laparoendosc. Adv. Surg. Tech..

[52] Esposito C., Coppola V., Del Conte F., Cerulo M., Esposito G., Farina A., Crocetto F., Castagnetti M., Settimi A., Escolino M. (2020). Near-Infrared fluorescence imaging using indocyanine green (ICG): Emerging applications in pediatric urology. J. Pediatr. Urol..

[53] Herz D., DaJusta D., Ching C., McLeod D. (2016). Segmental arterial mapping during pediatric robot-assisted laparoscopic heminephrectomy: A descriptive series. J. Pediatr. Urol..

[54] Esposito C., Autorino G., Coppola V., Esposito G., Paternoster M., Castagnetti M., Cardone R., Cerulo M., Borgogni R., Cortese G. (2021). Technical standardization of ICG near-infrared fluorescence (NIRF) laparoscopic partial nephrectomy for duplex kidney in pediatric patients. World J. Urol..

[55] Esposito C., Soria-Gondek A., Castagnetti M., Cerulo M., Del Conte F., Esposito G., Pecoraro C., Cicala D., Farina A., Escolino M. (2020). Laparoscopic or Robotic Deroofing Guided by Indocyanine Green Fluorescence and Perirenal Fat Tissue Wadding Technique of Pediatric Simple Renal Cysts. J. Laparoendosc. Adv. Surg. Tech..

[56] Carty K.N., Hwang A., Gordon A., Locke R., DeMarco R.T., Bayne C.E. (2021). Indocyanine green (ICG) assessment of ureteral perfusion during pediatric robotic surgery. J. Pediatr. Surg. Case Rep..

[57] Martins D.B., Farias-Eisner G., Mandelbaum R.S., Hoang H., Bradley J.P., Lee J.C. (2016). Intraoperative Indocyanine Green Laser Angiography in Pediatric Autologous Ear Reconstruction. Plast. Reconstr. Surg.-Glob. Open.

[58] Ishikawa K., Sasaki S., Furukawa H., Nagao M., Iwasaki D., Saito N., Yamamoto Y. (2013). Preliminary Experience With Intraoperative Near-infrared Fluorescence Imaging in Percutaneous Sclerotherapy of Soft-Tissue Venous Malformations. Dermatol. Surg..

[59] Fried F.W., Beier J.P., Bohr C., Iro H., Horch R.E., Arkudas A. (2019). Free Latissimus Dorsi Myocutaneous Flap in a 6-Month-Old Child for Reconstruction of a Temporal Fossa Defect After Teratoma Resection. Ann. Plast. Surg..

[60] Hinchcliff K.M., Yao A., Taub P.J. (2013). Laser-Assisted Indocyanine Green Imaging to Assess Perfusion of Scalp Closure in an Infant. J. Craniofacial Surg..

[61] Li S., Zhao Y., Zhang Y., Liao J., Hua K., Gu Y., Wang D., Tian J., Huang J. (2022). Indocyanine green localization for laparoscopic duodenal web excision. Photodiagnosis Photodyn. Ther..

[62] Paraboschi I., De Coppi P., Stoyanov D., Anderson J., Giuliani S. (2021). Fluorescence imaging in pediatric surgery: State-of-the-art and future perspectives. J. Pediatr. Surg..

[63] Privitera L., Waterhouse D.J., Preziosi A., Paraboschi I., Ogunlade O., Da Pieve C., Barisa M., Ogunbiyi O., Weitsman G., Hutchinson J.C. (2023). Short-wave infrared imaging enables high-contrast fluorescence-guided surgery in neuroblastoma. Cancer Res..

[64] Wellens L.M., Deken M.M., Sier C.F.M., Johnson H.R., de la Jara Ortiz F., Bhairosingh S.S., Houvast R.D., Kholosy W.M., Baart V.M., Pieters A.M.M.J. (2020). Anti-GD2-IRDye800CW as a targeted probe for fluorescence-guided surgery in neuroblastoma. Sci. Rep..

[65] Mulita F., Verras G.-I., Anagnostopoulos C.-N., Kotis K. (2022). A Smarter Health through the Internet of Surgical Things. Sensors.

